# Evolution of an archaeal virus nucleocapsid protein from the CRISPR-associated Cas4 nuclease

**DOI:** 10.1186/s13062-015-0093-2

**Published:** 2015-10-29

**Authors:** Mart Krupovic, Virginija Cvirkaite-Krupovic, David Prangishvili, Eugene V. Koonin

**Affiliations:** Department of Microbiology, Unité Biologie Moléculaire du Gène chez les Extrêmophiles, Institut Pasteur, Paris, 75015 France; National Center for Biotechnology Information, National Library of Medicine, National Institutes of Health, Bethesda, MD 20894 USA

**Keywords:** Virus evolution, Capsid proteins, Nucleocapsid, Virus origin, Archaea viruses

## Abstract

**Abstract:**

Many proteins of viruses infecting hyperthermophilic Crenarchaeota have no detectable homologs in current databases, hampering our understanding of viral evolution. We used sensitive database search methods and structural modeling to show that a nucleocapsid protein (TP1) of Thermoproteus tenax virus 1 (TTV1) is a derivative of the Cas4 nuclease, a component of the CRISPR-Cas adaptive immunity system that is encoded also by several archaeal viruses. In TTV1, the Cas4 gene was split into two, with the N-terminal portion becoming TP1, and lost some of the catalytic amino acid residues, apparently resulting in the inactivation of the nuclease. To our knowledge, this is the first described case of exaptation of an enzyme for a virus capsid protein function.

**Reviewers:**

This article was reviewed by Vivek Anantharaman, Christine Orengo and Mircea Podar. For complete reviews, see the Reviewers’ reports section.

**Electronic supplementary material:**

The online version of this article (doi:10.1186/s13062-015-0093-2) contains supplementary material, which is available to authorized users.

## Findings

The ability to form virions is the key feature which distinguishes viruses from other types of mobile genetic elements, such as plasmids and transposons [1–7]. The origin of *bona fide* viruses thus appears to be intimately linked to and likely concomitant with the origin of the capsids. However, tracing the provenance of viral capsid proteins (CPs) proved to be particularly challenging because they typically do not display sequence or structural similarity to proteins from cellular life forms [1, 8]. Over the years, a number of structural folds have been discovered in viral CPs. Strikingly, morphologically similar viral capsids, in particular, icosahedral, spindle-shaped and filamentous ones, can be built from CPs which have unrelated folds [8–11]. Thus, in the course of evolution, viruses have found multiple solutions to the same problem [12]. Nevertheless, the process of *de novo* origin of viral CPs remains largely enigmatic. Here we show that one of the capsid proteins of a filamentous archaeal virus, Thermoproteus tenax virus 1 (TTV1), evolved relatively recently through exaptation from a CRISPR-associated Cas4 nuclease.

**Fig. 1:**
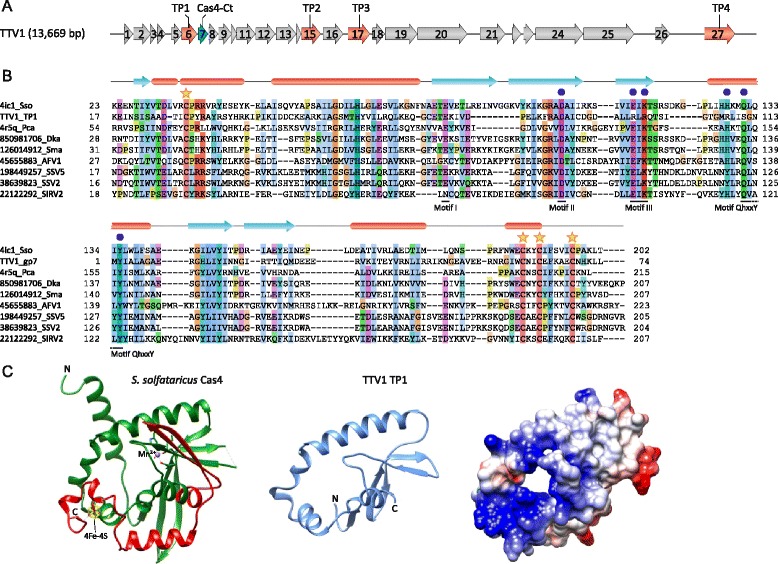
Analysis of TTV1 nucleocapsid protein TP1. **a** Genome map of TTV1 with the four genes encoding capsid proteins highlighted in red. The newly annotated open reading frame (ORF7), which encodes a putative protein corresponding to the C-terminal part of the Cas4 nuclease, is shown in cyan. **b** Multiple sequence alignment of TTV1 TP1 (*upper* block) and gp7 (*bottom* block) with Cas4 nucleases from archaea and their viruses. Sequences are indicated with GenBank or PDB identifiers. *Red* ellipses and *blue arrows* above the alignment respectively correspond to α-helices and β-strands experimentally determined for Cas4 protein (Sso0001) from *S. solfataricus* [26]. The four conserved cysteine residues coordinating the Fe-S cluster are indicated with stars, whereas amino acid residues conserved in the RecB-like nucleases and shown to be important for catalysis [26, 27] are indicated with blue circles. The conserved motifs are also highlighted under the alignment. **c** X-ray structure of the Cas4 nuclease from *S. solfataricus* (*left*) was used as a template to build a model of TTV1 TP1 (*middle* and *right*). In the Cas4 structure, the region shared with TP1 is colored green, whereas the C-terminal domain which corresponds to TTV1 gp7 is in red. Models in the middle and on the right are depicted using ribbon and surface rendering, respectively. The latter is colored according to the electrostatic surface potential following Coulomb’s law. The color scale is from −7 (*red*) to +7 (*blue*) kcal/(mol·e)

TTV1 was the first virus to be isolated from a hyperthermophilic archaeon [13]. It infects the crenarchaeon *Thermoproteus tenax* which grows optimally at 86 °C. The virions are flexible filaments 400 × 40 nm in size and consist of a linear, ~16 kb dsDNA genome (Fig. 1a), four capsid proteins (TP1-4) and a lipid-containing envelope [14]. Dissociation experiments have shown that basic proteins TP1 and TP2, which are present in equimolar amounts, interact with dsDNA genome and form the nucleocapsid of the virus [13–16]. None of the TTV1 capsid proteins shows recognizable sequence similarity to structural proteins of other archaeal filamentous viruses [17] and virion organization of TTV1 also appears to be unique [18]. This prompted us to re-evaluate the features of the structural proteins of TTV1 with the goal of understanding the provenance of this virus and its relationship to other viruses of archaea.

Analysis of the TP2, TP3 and TP4 sequences showed that despite the fact that over 30 years have passed since the TTV1 isolation these proteins remain to be ORFans without detectable homologs in other known viruses or cellular organisms. Although BLASTP searches [19] seeded with the TP1 sequence also failed to identify homologs in public databases, more sensitive Hidden Markov model-based HHpred analysis [20] resulted in a highly significant hit. Unexpectedly, TP1 was found to be homologous to Cas4 nuclease (PHA00619; *P* = 94.5, E = 7.8e-02), one of the proteins associated with the prokaryotic CRISPR-Cas immunity system [21, 22]. The match encompassed 70 % of the TP1 sequence (residues 11–90 out of 113) and corresponded to the N-terminal half of the Cas4 proteins (Additional file 1: Figure S1A). Notably, the hit to nucleases of the PD-(D/E)XK superfamily was considerably weaker (PF12705; *P* = 70.9). Multiple sequence alignment of TP1 with selected Cas4 proteins from cellular organisms and archaeal viruses has further extended the aligned region and confirmed the homology of these proteins (Fig. 1b). However, the C-terminal region that is conserved in Cas4 proteins remained unaccounted for. Additional analysis of the TTV1 genome showed that a region between nucleotides 1853 and 2074 (X14855), immediately downstream of the TP1 gene, encompasses a previously unannotated open reading frame (74 codons, denoted *7* in Fig. 1a) which corresponds to the missing C-terminal part of the Cas4 proteins. HHpred analysis initiated with the gp7 sequence returned multiple significant hits to Cas4 proteins (*P* = 96.3, E = 4.9e-03; Additional file 1: Figure S1B) and multiple sequence alignment is fully consistent with this result (Fig. 1b). Collectively, genes encoding TP1 and gp7 reconstitute a full-length Cas4 gene and the split in the ancestral TTV1 Cas4 has apparently occurred within the QhxxY motif that is conserved in the Cas4 family and other RecB-like nucleases (Fig. 1b). The experimentally determined molecular weight of TP1 (~14 kDa) [13] is consistent with the mass of this protein estimated from the sequence (12.9 kDa). The molecular weight of the reconstituted, full-length Cas4 would be considerably larger (21.6 kDa), confirming that the split between TP1- and gp7-encoding genes does not result from a sequencing error and that TP1 functions as a stand-alone protein. Although it is not known whether gp7 is expressed, this protein does not appear to be part of the TTV1 virion [13].

The Cas4 nuclease is widespread in Type I and Type II CRISPR-Cas systems and is believed to be involved in the acquisition of new spacers together with Cas1 and Cas2 proteins, and possibly additional CRISPR-associated functions [23–25]. Biochemical characterization of several Cas4 proteins has shown that they possess a broad spectrum of activities in vitro, including endonuclease, 5′ → 3′ and 3′ → 5′ exonuclease as well as ATP-independent DNA unwinding activities [26, 27]. High-resolution X-ray structures of two Cas4 proteins have been solved – one from *Sulfolobus solfataricus* [26] and the other from *Pyrobaculum calidifontis* [27]. The proteins consist of two domains the N-terminal RecB-like nuclease domain and the C-terminal domain containing a Fe-S cluster coordinated by 4 conserved cysteine residues [26, 27] (Fig. 1c). Notably, the 4 conserved Cys residues are split between TP1 and gp7, with Cys1 located within TP1 and the 3 remaining Cys in gp7. Furthermore, not all active site residues characteristic of the RecB-like nucleases [26, 27] are preserved in TP1; in particular, the glutamate residue located in Motif III and involved in the coordination of a metal ion is replaced by an arginine in TP1 (Fig. 1b). Thus, TP1 lacks the Fe-S cluster and is unlikely to be catalytically active due to mutations in the active site, including the truncation of the Motif QhxxY.

To better assess the implications of the changes described above for the function of TP1, we built its structural model (Fig. 1c) based on the X-ray structure of Cas4 from *S. solfataricus* (Sso0001, PDB ID: 4ic1; [26]; the two proteins are 24 % identical within the aligned regions). Analysis of the electrostatic charge distribution in TP1 model revealed a highly positively charged surface encompassing the N-terminus of the protein, which might be important for DNA binding. Importantly, the corresponding surface in Cas4 is shielded by the C-terminal domain, suggesting that removal of this domain was a prerequisite for the transformation of the ancestral TTV1 Cas4 into a nucleocapsid protein.

## Concluding remarks

Cas4 nuclease is a conserved component of the CRISPR-Cas systems. Beyond CRISPR-Cas, Cas4-like nucleases are also occasionally encoded in casposons, a recently discovered group of large transposable elements [28], as well as genomes of bacterial and archaeal viruses [29–31]. Among archaeal viruses, Cas4-like proteins are encoded by members of at least three different families, including *Rudiviridae*, *Lipothrixviridae* and *Fuselloviridae* (Fig. 1b), and in the case of rudivirus SIRV2, the protein has been shown to possess both 5′ → 3′ exonuclease and endonuclease activities in vitro [29, 32]. Thus, it appears likely that the ancestor of TTV1 encoded a functional Cas4 nuclease which could participate in certain aspects of genome replication or repair. At a certain point in evolution, the gene was truncated, possibly following the mutation(s) in the active site, and the region encoding the N-terminal domain of the Cas4 nuclease evolved into a DNA-binding protein which was recruited as a new nucleocapsid protein of TTV1. Exaptation, a process whereby a function or a trait changes during evolution, is a major evolutionary phenomenon [33] that has also contributed to the evolution of viruses [34, 35]. Our finding that a Cas4-like nuclease has evolved into a viral CP provides another striking example of exaptation in virus evolution. More generally, it appears that viral CPs can occasionally evolve from functionally diverse proteins that originally had no involvement in formation of virus-like particles.

## Methods

The non-redundant database of protein sequences at the NCBI was searched using the PSI-BLAST [19]. Protein sequences were aligned with Promals3D [36]. The alignment was visualized using Jalview [37]. Profile-against-profile searches were performed using HHpred [20] against different protein databases, including PFAM, PDB, CDD, and COG, which are available via the HHpred website. The hit to Cas4 profile (PHA00619; *P* = 94.5) was obtained when the search was performed against the CDD database (the alignment of the hit is shown in the Additional file 1: Figure S1A). Structural modelling was done with Modeller v9.15 [38]. The resultant model (DOPE score of −14074.4) was then verified for stereochemical consistency using ProSA-web [39]; the Z-score was found to be −3.33. The TP1 structural model and the template (PDB ID: 4ic1) structures were visualized using UCSF Chimera [40].

## Reviewers’ reports

### Reviewer 1: Vivek Anantharaman (National Center for Biotechnology Information, National Library of Medicine, National Institutes of Health)

This is a straight forward paper describing that TP1 belongs to the Cas4 fold. I have a few questions and comments.

1) Line 72–74 When the TP1 protein by itself is run through HHPRED it only hits a member of the REase (PDDEXK) fold (PF12705). Only when the C terminal gp7 sequence is added does the hit to Cas4 stand out. This needs to be clarified.

Authors’ response: *We performed HHpred searches against different databases accessible though the HHpred website, including PFAM, PDB (Protein Data Bank), CDD (Conserved Domain Database at NCBI), and COG (clusters of orthologous genes). This information has now been added to the Methods section. The hit to Cas4 profile (PHA00619; P = 94.5) was specifically obtained when the search was performed against the CDD database (the alignment of the hit is shown in the* Additional file 1: Figure S1A*). The hit to PDDEXK-like nucleases was considerably weaker (P = 70.9). This is now clarified in the revised manuscript.*

2) Is there evidence that the gp7 protein (the C terminal part) is not being made? If so please mention it. If not, the two proteins could function together as subunits and at least retain the metal binding role.

Authors’ response: *Unfortunately, the information regarding gp7 expression is not available. However, even if the protein is expressed, it does not appear to be part of the TTV1 virion. We point this out in the revised manuscript.*

3) Only the E among the conserved active site residues is changed to R. There are instances of REases that are active with changes to the E and Ref 25 shows QxxxY mutants retain their activity. The paper rightly says “unlikely” to be active in Line:105, but becomes more emphatic in attributing inactivity in the conclusion. Unless experimentally verified, one can only say it is most likely to be inactive.

Authors’ response: *We agree with the reviewer that caution should be exercised and removed the claim regarding the lack of nuclease activity from the Conclusions section.*

Minor issues

3) The conserved H is also part of the active site (ref 25,26) and is not marked in the figure.

Authors’ response: *The conserved His is now also highlighted.*

### Reviewer 2: Christine Orengo (Institute of Structural and Molecular Biology, University College London)

The article presents an interesting analysis of the evolutionary relationship between a viral capsid protein and the Cas4 nuclease family. Capsid proteins have been found to derive from a range of different fold groups and this paper shows the recruitment of a protein with another type of structure, originally functioning as an enzyme, to a new function as a capsid protein. Sound methodology has been used but the authors should include more details on the significance of the matches they detect.

This is a brief but interesting paper demonstrating an evolutionary relationship between a viral capsid protein and a Cas4 nuclease. The TTV1 capsid protein studied has no significant sequence similarity to other archaeal virus proteins and the virion organisation is unique. The authors have detected this relationship using a well-established method, HHpred, which is probably the most powerful method available to date for identifying distant realtionships. Analysis of a multiple alignment generated using the sequences identified by the searches shows that the capsid protein has lost the catalytic residues necessary for the nuclease activity. A 3D model built from an available structure of Cas4 nuclease reveals a highly positively charged patch which may be implicated in DNA binding. This region is concealed by the C-terminal domain in Cas4 and the authors speculate that the removal of the C terminal region - detected by their studies - is necessary for the transformation of the Cas4 nuclease into a nucleocapsid protein. The manuscript summarises the literature on these proteins well and presents interesting observations but it would have been helpful to have more details of the results of the computational work. For example what E-value does HHpred give for the match ie how significant is the match.

Authors’ response: *The significance scores were provided in the* Additional file 1: Figure S1*. The HHpred probabilities of the TP1 and gp7 hits to Cas4 were 94.53 (E-value = 0.078) and 96.31 (E-value = 0.0049), respectively. These values are now also indicated in the main text.*

Similarly, what is the sequence similarity between the capsid protein and the Cas4 nuclease structure used to model it and what is the quality of the model built (eg DOPE score). This data should be given in the manuscript as it is necessary to judge the validity of the authors conclusions.

Authors’ response: *The sequence identity between TP1 and the template structure, the DOPE score as well as the ProSA-Web score, which was used to further verify the stereochemical consistency of the model, are now provided in the main text and the Methods section.*

Minor issues

Details of the sequence search (eg E-value of the match) should be presented in the main text.

Authors’ response: *All this information is now provided.*

### Reviewer 3: Mircea Podar (Biosciences Division, Oak Ridge National Laboratory)

This is a very interesting finding about exaptation of Cas4 to serve as a structural viral protein. The article is well written and I have no criticism to the approach and data interpretation. This finding is important in the quest to understanding the evolution of viruses and how structural elements in capsids diversify across evolutionary distances.

It seems that only one strain of TTV1 is available, is that right? If so, are there perhaps TTV-type sequences present in metagenomic datasets? It would be valuable if such closely related viruses or viral genomes would be available as they would provide information on more recent changes/variants of the TP1, even finding variants in which the original enzymatic function of Cas4 may still be present. It is unclear if the HHPred search was also performed against metagenomic sequences.

Authors’ response: *Finding the intermediates with different variants of Cas4/TP1 genes would be indeed interesting. Unfortunately, no other TTV1-like viruses have been described thus far and, to the best of our knowledge, sequences closely matching TTV1 have never been reported in the metagenomics studies. Collection of all available metagenomes and their assembly into contigs is a considerable effort, which appears to be beyond the scope of the current short report.*

## Additional file

Additional file 1: Figure S1. Results of the HHpred search seeded with TP1 (A) and gp7 (B) sequences. H(h), α-helix; E(e), β-strand; C(c), coil. (PDF 237 kb)

## Abbreviations

AFV1: Acidianus filamentous virus 1 (*Lipothrixviridae*)
BLAST: basic local alignment search tool
CDD: conserved domain database
COG: clusters of orthologous genes
CP: capsid protein
CRISPR-Cas: Clustered Regularly Interspaced Short Palindromic Repeats/CRISPR-associated proteins
Dka: *Desulfurococcus kamchatkensis*
DOPE: discrete optimized protein energy
Pca: *Pyrobaculum calidifontis*
PDB: protein data bank
SIRV2: *Sulfolobus islandicus* rod-shaped virus 2 (*Rudiviridae*)
Sma: *Staphylothermus marinus* F1
Sso: *Sulfolobus solfataricus*
SSV2 and SSV5: *Sulfolobus* spindle-shaped viruses 2 and 5 (*Fuselloviridae*)
TTV1: *Thermoproteus tenax* virus 1
