# Supplementary material for: Evolution of an archaeal virus nucleocapsid protein from the CRISPR-associated Cas4 nuclease
Source: Biol Direct. 2015 Oct 29;10:65. doi: 10.1186/s13062-015-0093-2 (PMC4625639; doi:10.1186/s13062-015-0093-2)
Supplement: Additional file 1: Figure S1. — Results of the HHpred search seeded with TP1 (A) and gp7 (B) sequences. H(h), α-helix; E(e), β-strand; C(c), coil. (PDF 237 kb) [file 13062_2015_93_MOESM1_ESM.pdf]

A.

```
>PHA00619 CRISPR-associated Cas4-like protein
  Probab=94.53  E-value=0.078  Score=42.16  Aligned_cols=78  Identities=27%  Similarity=0.323  Sum_probs=0.0

  Q ss_pred              eeccCCcccccccccc-ccCchHHHHHhhhcC-CcccchhHHHHHhcccchhHHHHHHHHHcCCcccccc------Chhh
  Q Wed_Aug_26_18:    11 YTIDENKEINSISAAD-TICPYRAYRSYHRKI-PIKIDDCKEARKIAGSMTHYVILRQLKEQGCETEKIV------DPEL   82 (113)
  Q Consensus         11 ytidenkeinsisaad-ticpyrayrsyhrki-pikiddckearkiagsmthyvilrqlkeqgcetekiv------dpel   82 (113)
                         |.+-+.+..+.|-+.| ..||-++|  |-++. |.+++.-.--+-+.|+..|-.+-+-|++.|.|+|+.|      +.++
  T Consensus         12 ~~~~~~~~~~~i~vTdl~~CpRk~~--~~~~~~~~~~~~~~~~~~~~G~~lHe~le~iLk~~g~e~E~eve~~~~~g~~I   89 (201)
  T PHA00619          12 YEIVSQNDLQTIWVTELSRCLRRSW--LMRKNGGVKLALEEAMKMHIGSGLHMRLQRILKKHGFETECRVERKTALGFEI   89 (201)
  T ss_pred              HhhcccCCCCEEEEeehhhCccHHH--HHHhCCcccccchhhHHHHhhHHHHHHHHHHHHHhCCceeEEEEEEEcCCCcEE


  Q ss_pred              cccccccc
  Q Wed_Aug_26_18:    83 KFRADAIC   90 (113)
  Q Consensus         83 kfradaic   90 (113)
                         +-|+|+++
  T Consensus         90 ~GrID~i~   97 (201)
  T PHA00619          90 VGKIDVYD   97 (201)
  T ss_pred              EEEEEEEe
```

B.

```
>4ic1_A CAS4, crispr, MCSG, exonuclease, PSI-biology, structural GEN unknown function; 2.35A {Sulfolobus solfataricus}
  Probab=96.31  E-value=0.0049  Score=40.81  Aligned_cols=67  Identities=24%  Similarity=0.339  Sum_probs=0.0

  Q ss_pred              eEEecccccccEEEEcCCeeEEEEcChHHHhhHHHHHHHHHHHhhCchhhhhhcCceeeehhhhhhhhcccc
  Q Thu_Aug_27_19:     2 YIALAGAERGHLIYINNGIRTTIQMDEEERVKITEYVRNLIRRIKNGEAVEERNRGIWCNICEFKAECNHKL   73 (74)
  Q Consensus          2 yialagaerghliyinngirttiqmdeeervkiteyvrnlirrikngeaveernrgiwcnicefkaecnhkl   73 (74)
                         |..+.....|-|+|++.+-...+..|++.+.+.   +.+.|+.|.++....  ..+-+|.-|.|+.-|..+.
  T Consensus        139 Y~~~~~~~~g~l~y~~~~~~~~~~~~~~~~~~~~~---l~~~i~~i~~~~~~P--~~~~~C~~C~~~~~C~~~~   205 (206)
  T 4ic1_A          139 YLWLFSAEKGILVYITPDRIAEYEINEPLDEAT---IVRLAEDTIMLQNSP--RFNWECKYCIFSVICPAKL   205 (206)
  T ss_dssp              HHHHHTCSEEEEEEEETTEEEEEEECCCCCHHH---HHHHHHHHHTTCSSS--SSTTSGGGCTTGGGCTTCC
  T ss_pred              HHHHHhccCcEEEEEeCCceEEEecCcchhHHH---HHHHHHHHHhcCCCC--CCCCCCCCCCCccccCCcc


>PHA00619 CRISPR-associated Cas4-like protein
  Probab=93.46  E-value=0.14  Score=38.31  Aligned_cols=67  Identities=19%  Similarity=0.303  Sum_probs=0.0

  Q ss_pred              eEEecccccccEEE-EcCCeeEEEEcChHHHhhHHHHHHHHHHHhhCchhhhhhc-Cceeeehhhhhhhh
  Q Thu_Aug_27_19:     2 YIALAGAERGHLIY-INNGIRTTIQMDEEERVKITEYVRNLIRRIKNGEAVEERN-RGIWCNICEFKAEC   69 (74)
  Q Consensus          2 yialagaerghliy-inngirttiqmdeeervkiteyvrnlirrikngeaveern-rgiwcnicefkaec   69 (74)
                         |+-+++++.|.|+| --++..-.|. +++....+.+-+..+.+-+++-..-+..- ...+|..|.|+.-|
  T Consensus        129 Yl~~l~~~~G~L~~V~~d~~~e~v~-~d~~e~el~~~~~~l~~~l~~~~~p~~~p~~~~~C~~C~f~~~C   197 (201)
  T PHA00619        129 YIEMANAMAGYLIIVHADGRVEEIK-RDWSETDLENRANAFGISVEENILPPKKSKPDSECIECPFYNVC   197 (201)
  T ss_pred              HHHHHHHcCCEEEEEeCCCCeEEec-CchhHHHHHHHHHHHHHHHhccCCCCCCCCcCCCCCCCCCChhhc
```

Figure S1. Results of the HHpred search seeded with TP1 (A) and gp7 (B) sequences. H(h), α-helix; E(e), β-strand; C(c), coil.
